# Processing Status, Rather Than Meat Source, Is Associated with Metabolic Dysfunction–Associated Steatotic Liver Disease Among US Adults

**DOI:** 10.5152/tjg.2026.26294

**Published:** 2026-07-09

**Authors:** Shan Qin, Zhaowei Li, Fuman Yang, Qian Feng, Rui Wang, Zhong Chen

**Affiliations:** Department of Ultrasound, The General Hospital of Western Theater Command, Sichuan, China

**Keywords:** Controlled attenuation parameter, NHANES, MASLD, processed meat, unprocessed meat

## Abstract

**Background/Aims:**

Metabolic dysfunction–associated steatotic liver disease (MASLD) is one of the most common chronic liver diseases worldwide and is closely related to modifiable dietary factors. The authors aimed to examine whether the association between meat intake and MASLD is more closely related to meat source or processing status.

**Materials and Methods:**

The current study analyzed data from 1828 adults in the 2017-2018 National Health and Nutrition Examination Survey (NHANES). Meat intake was assessed using 24-hour dietary recalls and classified into processed red meat, processed poultry, unprocessed red meat, and unprocessed poultry. The presence of MASLD was determined using the controlled attenuation parameter measured by vibration-controlled transient elastography.

**Results:**

In fully adjusted models, each 1 oz-eq/day increase in processed meat was associated with 27% higher odds of MASLD (odds ratio (OR) 1.27, 95% CI 1.10-1.46, *P* = .003). Patients in the highest tertile had 2.46 times higher odds of MASLD than those in the lowest tertile (*P* = .002), with a robust linear trend (*P* for nonlinearity = .817). This association was observed for both processed red meat and processed poultry. In contrast, neither total unprocessed meat nor its specific subtypes (unprocessed red meat and unprocessed poultry) was significantly associated with MASLD. Iso-serving substitution analysis indicated that replacing 1 oz-eq/day of processed meat with unprocessed meat was associated with a 24% reduction in the odds of MASLD (OR 0.76, 95% CI 0.66-0.88; *P* = .001).

**Conclusion:**

The observed association with MASLD appeared to be more closely related to processing status than to meat source.

**Main Points**

- Higher intake of processed meat, including both processed red meat and processed poultry, was independently associated with higher odds of metabolic dysfunction–associated steatotic liver disease (MASLD).
- In contrast, the consumption of unprocessed meat, whether red meat or poultry, was not significantly associated with MASLD.
- These findings suggest that processing-related characteristics may be more relevant than meat source to the observed hepatometabolic association.
- Theoretical substitution modeling suggests that replacing processed meat with an equivalent amount of unprocessed meat was associated with lower odds of MASLD.

## Introduction

Metabolic dysfunction–associated steatotic liver disease (MASLD) is one of the most common chronic liver disorders worldwide and is closely associated with obesity, insulin resistance, type 2 diabetes, and cardiovascular disease.^1^^-^^5^ Given the lack of widely accepted pharmacologic therapy for most patients, lifestyle-based prevention and management remain central to current clinical practice.^6^ Among modifiable lifestyle factors, diet is particularly important because it influences hepatic lipid accumulation, systemic inflammation, and metabolic homeostasis.

Dietary patterns rich in refined carbohydrates, ultra-processed foods, and saturated fat are associated with hepatic steatosis, whereas healthier dietary patterns such as the Mediterranean diet appear beneficial.^7^^,^^8^ Among specific food groups, processed meat consumption has attracted particular concern.^9^ Higher processed meat intake is consistently associated with increased risks of type 2 diabetes and coronary heart disease in large meta-analyses.^10^^,^^11^ Proposed biological mechanisms include high sodium content, nitrates and nitrites, heme iron, and saturated fat, which may promote oxidative stress, inflammation, and insulin resistance, all of which are implicated in hepatic steatosis.^12^^-^^14^

Despite these mechanistic plausibilities and established cardiometabolic associations, direct epidemiological evidence linking processed meat intake specifically to MASLD remains limited. Several important gaps warrant further investigation. First, most epidemiological studies examined “red and processed meat” as a combined exposure, without distinguishing processed and unprocessed meats by specific source subtypes (red meat versus poultry). By failing to fully disentangle these categories, it remains unclear whether observed hepatometabolic associations are driven by the meat source or by artificial processing methods. These subtypes differ in nutrient composition and heme iron content, which may differentially influence metabolic risk.^15^ A standardized method to disaggregate meat intake into high-resolution categories (including processed red meat, processed poultry, unprocessed red meat, and unprocessed poultry) using the Food Patterns Equivalents Database (FPED) was recently developed, improving exposure precision in population-based dietary analyses.^15^ Nevertheless, this granular classification has rarely been applied in studies examining liver-related outcomes. Second, many prior investigations of fatty liver disease have relied on surrogate indices such as the fatty liver index or liver enzyme levels, which may introduce outcome misclassification.^16^ Controlled attenuation parameter (CAP), measured by ultrasound transient elastography, provides a validated, noninvasive assessment of hepatic steatosis and is increasingly used in clinical and epidemiological research.^17^ Use of CAP-defined MASLD may therefore improve the validity of observed associations. Third, limited evidence exists regarding dose–response relationships and potential substitution effects in the association between processed meat and MASLD. Modeling the theoretical replacement of processed meat with unprocessed meat provides clinically relevant insights into dietary modification strategies and reflects real-world dietary tradeoffs.

The current study was designed to address these gaps. Using nationally representative data from the 2017-2018 National Health and Nutrition Examination Survey (NHANES), the current study examined whether CAP-defined MASLD was differentially associated with processed and unprocessed meat intake after further categorizing meat by source (red meat vs poultry). The analysis also evaluated whether the association between processed meat and MASLD demonstrated a dose–response relationship and whether theoretical substitution of processed meat with unprocessed meat was associated with lower odds of MASLD. By distinguishing processing status from source subtype, the current study aimed to clarify which dimension of meat intake is more strongly associated with MASLD.

## Materials and Methods

### Study Design and Participants

This cross-sectional study used data from the 2017-2018 NHANES. CAP, measured by ultrasound transient elastography, has been available in NHANES since 2017. Standardized processed meat disaggregation variables were available for survey cycles from 2017 to 2020. However, the 2019-March 2020 cycle was discontinued early because of the COVID-19 pandemic and did not represent a complete 2-year survey cycle. To ensure methodological consistency, data completeness, and appropriate survey weighting, the analysis was restricted to the complete 2017-2018 cycle.

**Figure 1.**
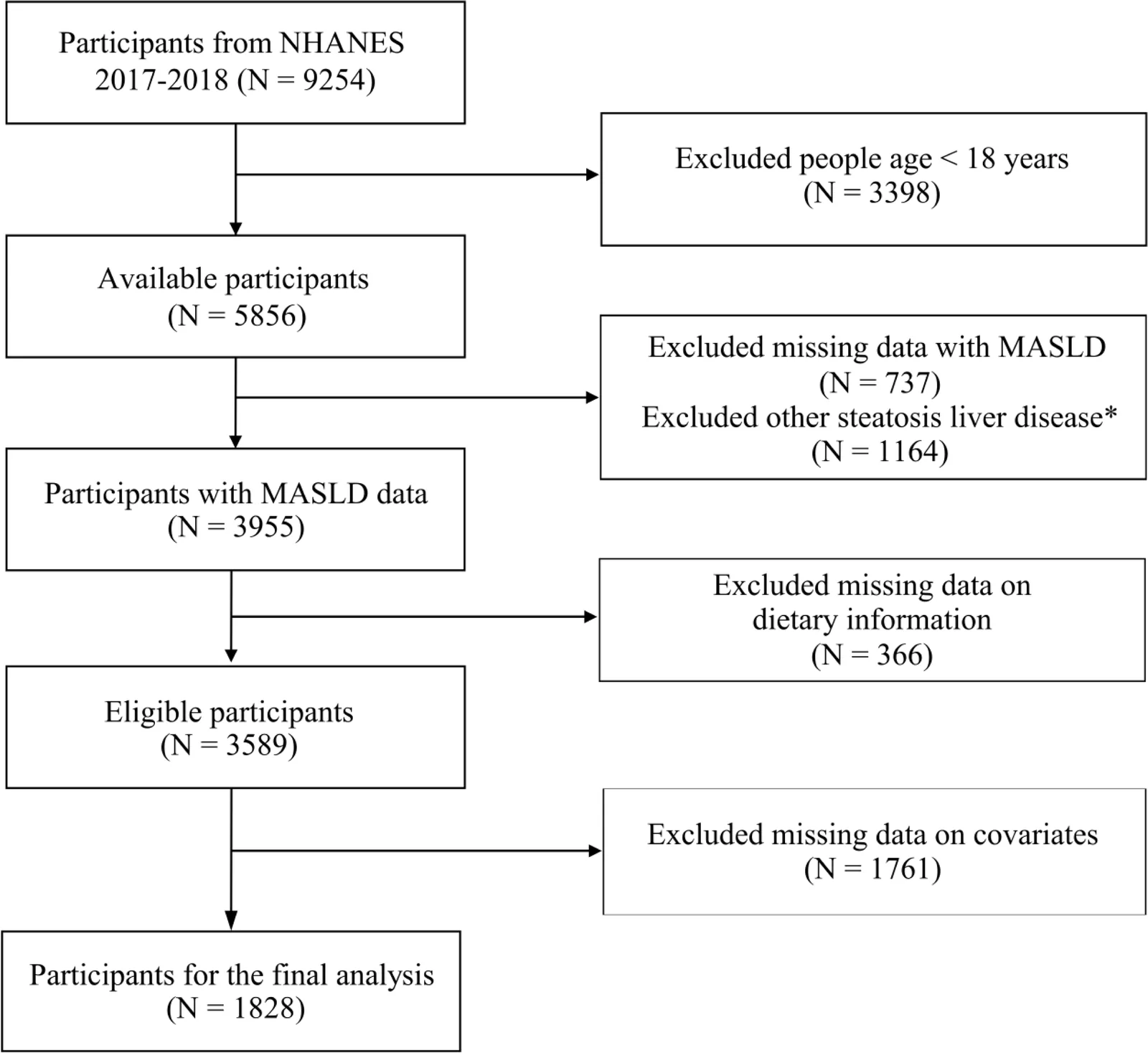
Participant selection and inclusion. Other steatotic liver disease (SLD) was defined as metabolic associated alcoholic liver disease, cryptogenic SLD or other combination etiology SLD, and other SLD due to specific etiologies.

As shown in Figure 1, adults aged 18 years or older were eligible for inclusion. Participants were excluded if they lacked CAP measurements, had alternative causes of steatotic liver disease, had missing dietary exposure data, or had incomplete covariate information. After application of these criteria, 1828 participants were included in the analysis. This study was conducted and reported in accordance with the Strengthening the Reporting of Observational Studies in Epidemiology guideline.

Ethics approval and written informed consent for data collection were obtained by NHANES. Specifically, the 2017-2018 NHANES cycle was approved by the National Center for Health Statistics Research Ethics Review Board under Protocol #2018-01, and written informed consent was obtained from all participants. Because the current study was a secondary analysis of publicly available, deidentified NHANES data, additional ethics committee approval and informed consent were not required at the authors’ institution.This study used ChatGPT (GPT-5.5 Thinking; OpenAI, San Francisco, CA, USA) to improve language and edit grammar during the revision of the manuscript. However, all content of the study has been reviewed and edited by the author(s) following the use of AI/LLM tools.

### Assessment of Processed Meat Intake

Processed meat intake was assessed using 24-hour dietary recall data from the 2017-2018 NHANES cycle, collected through the What We Eat in America (WWEIA) component using the USDA Automated Multiple-Pass Method. Dietary intake data were linked to the FPED, which converts reported foods into standardized food pattern components expressed as ounce-equivalents (oz-eq). To improve exposure precision, processed meat was disaggregated into processed red meat and processed poultry using a standardized method based on the Processed Meat Categories algorithm developed by O’Connor et al.^15^ Briefly, the FPED “cured meat” variable was reclassified as processed red meat and processed poultry according to food code descriptions and ingredient information from the Food and Nutrient Database for Dietary Studies. Chicken nuggets, patties, and fillets (WWEIA category 2204) were additionally classified as processed poultry because these items are considered further processed under some definitions and were included in the standardized Processed Meat Categories algorithm to improve capture of processed poultry intake.^15^

The primary exposure variable, total processed meat intake, was defined as the sum of pf_curedmeat (FPED cured meat component) and nug_pat_fil (chicken nuggets, patties, and fillets). Processed red meat intake was represented by cured_redmeat, and total processed poultry intake was represented by total_proc_poultry. Total unprocessed meat intake was defined as the sum of: pf_meat (unprocessed red meat) and unproc_poultry (unprocessed poultry). These 2 disaggregated subtypes were also analyzed independently as primary comparative exposures. Detailed information on the categorization and construction of these dietary meat variables is provided in the original methodological publication.^15^ Total processed meat intake was analyzed as both a continuous variable (per 1 oz-eq/day increment) and a categorical variable based on tertiles. Based on the population distribution (Supplementary Table 1), tertile cutoffs for total processed meat intake were defined as T1 (0 oz-eq/day), T2 (>0 to 1.015 oz-eq/day), and T3 (>1.015 oz-eq/day). Processed red meat, total unprocessed meat, unprocessed red meat, and unprocessed poultry were similarly categorized into weighted tertiles, whereas processed poultry was analyzed as a binary variable (0 vs. >0 oz-eq/day) because of its highly skewed distribution.

### Definition of Metabolic Dysfunction–Associated Steatotic Liver Disease

MASLD was defined using CAP, measured by ultrasound transient elastography. CAP provides a noninvasive quantitative assessment of hepatic steatosis and is expressed in decibels per meter (dB/m). Consistent with previous NHANES-based studies and established diagnostic thresholds, hepatic steatosis was defined as a CAP value of 248 dB/m or greater. Patients meeting this criterion were further classified as having MASLD if metabolic dysfunction was present and other causes of hepatic steatosis were excluded.

Other causes of steatotic liver disease were excluded based on available NHANES data and included: (1) excessive alcohol consumption; (2) hepatitis B or hepatitis C virus infection; (3) use of pharmacologic agents associated with hepatic steatosis (e.g., methotrexate, amiodarone, valproic acid, tamoxifen, glucocorticoids, and related medications); and (4) evidence of iron overload. MASLD was treated as a binary outcome variable (yes/no) in all analyses.

### Definition of Covariates

Socioeconomic variables included poverty income ratio (PIR), educational attainment, and marital status. Demographic variables included age, sex, and race/ethnicity. Lifestyle variables included smoking status, alcohol consumption, and physical activity. Physical activity was assessed as total metabolic equivalent minutes per week (MET-min/week). Total energy intake (kcal/day), derived from dietary recall data, was included in multivariable models to account for overall caloric intake. Overall diet quality was additionally assessed using the Healthy Eating Index-2015 (HEI-2015) and included in supplementary analyses. The derivation of HEI-2015 has been described previously.^18^

Metabolic comorbidities included diabetes mellitus, hypertension, and hyperlipidemia. Detailed diagnostic definitions and classification procedures are provided in Supplementary Table 2. High-density lipoprotein cholesterol (HDL-C) and serum triglycerides (TG) were additionally included as continuous variables.

### Statistical Analyses

For the primary analyses based on the average of 2 24-hour dietary recalls, the 2-day dietary sample weight (WTDR2D) was applied. In sensitivity analyses restricted to the first 24-hour dietary recall, the corresponding day 1 dietary weight (WTDRD1) was used to maintain national representativeness. Survey-weighted logistic regression was used to estimate odds ratios (ORs) and 95% CIs. As a supplementary analysis, the primary models were further adjusted for HEI-2015 to evaluate the potential influence of overall diet quality. In additional analyses, CAP and liver stiffness measurement (LSM) were analyzed as continuous outcomes using survey-weighted linear regression models, and advanced fibrosis was analyzed as a binary outcome using survey-weighted logistic regression models. Advanced fibrosis was defined as LSM ≥ 9.7 kPa.

Restricted cubic spline models were fitted to examine potential nonlinear dose–response relationships. Iso-serving substitution analyses were conducted by simultaneously including total meat intake and unprocessed meat intake in the same multivariable model, thereby estimating the association of replacing processed meat with an equivalent amount of unprocessed meat, while holding total meat intake constant. All analyses were performed using R software (version 4.3.1) (R Foundation for Statistical Computing; Vienna, Austria), and a *P* value of less than .05 was considered statistically significant.

## Results

### Baseline Characteristics of the Study Population

Table 1 summarizes the baseline characteristics of the 1828 participants, of whom 825 had MASLD. The prevalence of MASLD was significantly higher in males than in females (46.11% vs. 34.30%; *P* = .003) and increased progressively with age, peaking at 57.11% in individuals aged 60 years or older. As expected, participants with MASLD exhibited a poorer overall metabolic profile, characterized by a markedly higher prevalence of obesity (69.64%), diabetes (79.49%), hypertension, and hyperlipidemia, as well as elevated TG and reduced HDL-C (all *P* < .001). Furthermore, participants with MASLD engaged in significantly less physical activity (*P* = .001) than those without MASLD. No significant differences were observed in race/ethnicity, education levels, or PIR. Individuals with MASLD consumed significantly more total energy (2279 vs. 2128 kcal/d, *P* = .043), total processed meat (1.22 vs. 0.94 oz-eq/day, *P* = .032), and specifically processed red meat (0.81 vs. 0.53 oz-eq/day, *P* = .010). In contrast, the consumption of processed poultry, total unprocessed meat, unprocessed red meat, and unprocessed poultry did not differ significantly between the groups.

### Association Between Dietary Meat Intake and Metabolic Dysfunction–Associated Steatotic Liver Disease

Table 2 presents the survey-weighted multivariable logistic regression analyses evaluating the associations between dietary meat intake and odds of MASLD. In the fully adjusted model (Model 2), higher total processed meat intake was independently associated with greater odds of MASLD. When analyzed as a continuous variable, each 1 oz-eq/day increase in total processed meat intake was associated with a 27% higher odds of MASLD (OR 1.27, 95% CI 1.10-1.46; *P* = .003). This positive association was strongly supported by the categorical analysis. Compared with participants in the lowest tertile (T1) of total processed meat consumption, those in the highest tertile (T3) had significantly higher odds of MASLD (OR 2.46, 95% CI 1.48-4.09; *P* = .002). A significant dose–response relationship was also observed across the tertiles (*P* for trend = .025). Further disaggregation of processed meat subtypes revealed consistent detrimental effects for both red meat and poultry. In the fully adjusted models, each 1 oz-eq/day increase in processed red meat was associated with 23% higher odds of MASLD (OR 1.23, 95% CI 1.03-1.48; *P* = .028). Similarly, continuous intake of processed poultry was significantly associated with higher odds of MASLD (OR 1.35, 95% CI 1.10-1.65; *P* = .006). In contrast, total unprocessed meat intake was not significantly associated with MASLD. In the fully adjusted model, neither the continuous evaluation (OR 0.95, 95% CI 0.86-1.05; *P* = .334) nor the comparison of highest versus lowest tertile (OR 0.86, 95% CI 0.49-1.48; *P* = .557) was statistically significant. In the fully adjusted models, neither unprocessed red meat (continuous OR 1.02, 95% CI 0.92-1.12;* P* = .747) nor unprocessed poultry intake (continuous OR 0.93, 95% CI 0.81-1.06; *P* = .262) was significantly associated with MASLD.

### Subgroup and Sensitivity Analyses

**Figure 2.**
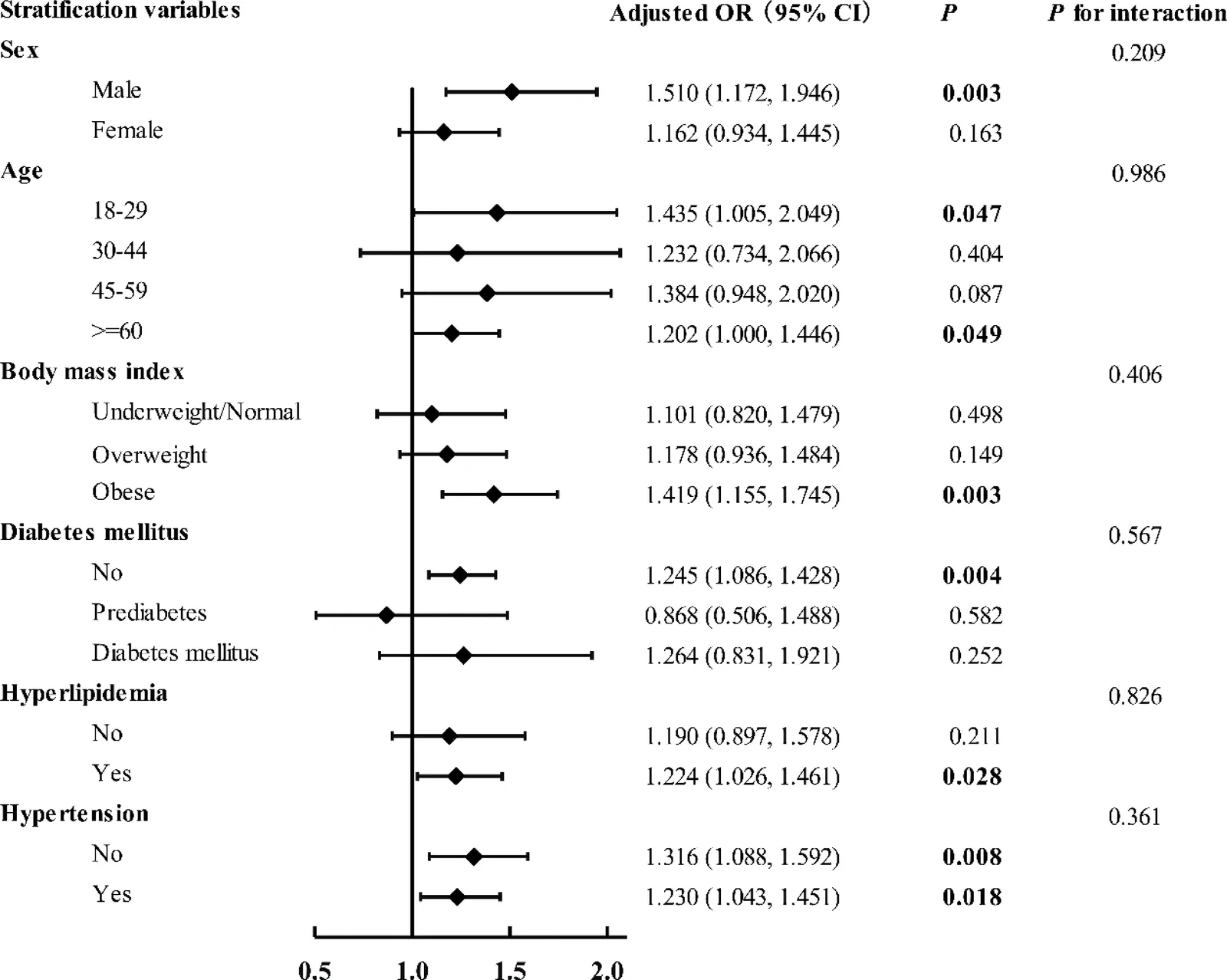
Adjusted association of processed meat (continuous) intake with metabolic dysfunction–associated steatotic liver disease for subgroup analyses. CI, confidence interval; OR, odds ratio.

To evaluate the robustness of the findings and identify potential effect modifiers, subgroup and sensitivity analyses were performed. Subgroup analyses (Figure 2) demonstrated that the positive association between continuous processed meat intake and MASLD risk remained largely consistent across various demographic and clinical subgroups, including age, sex, BMI categories, and the presence of metabolic comorbidities (all *P* for interaction > .05).

Furthermore, a sensitivity analysis using only the first 24-hour dietary recall data (Table 3) was performed to minimize potential reporting bias associated with the second telephone-based dietary interview. The results were consistent with the primary analyses. Higher processed meat intake remained significantly and independently associated with higher odds of MASLD in the fully adjusted models, whereas unprocessed meat intake remained unassociated with MASLD across all subtypes.

### Supplementary Analyses

To further evaluate the robustness and clinical relevance of the primary findings, several supplementary analyses were performed. First, after additional adjustment for HEI-2015, the positive association between processed meat intake and MASLD remained materially unchanged (Supplementary Table 3). Second, when CAP was analyzed as a continuous outcome, associations were attenuated after full adjustment, including adjustment for HEI-2015, suggesting that the observed relationship may be more evident for binary MASLD classification than for linear variation across the full CAP range (Supplementary Table 4). Third, analyses using LSM as a continuous outcome showed weak, nonsignificant associations after full adjustment (Supplementary Table 5). Finally, analyses for advanced fibrosis showed patterns that differed from the primary MASLD findings and should be interpreted with caution. Given the limited number of advanced fibrosis cases relative to the number of covariates in the fully adjusted model, these results were considered exploratory rather than definitive (Supplementary Table 6). Taken together, these supplementary analyses suggest that the association between processed meat intake and binary MASLD was relatively robust, whereas associations with continuous CAP, continuous LSM, and advanced fibrosis were weaker or inconsistent.

### Dose–Response Relationship

**Figure 3.**
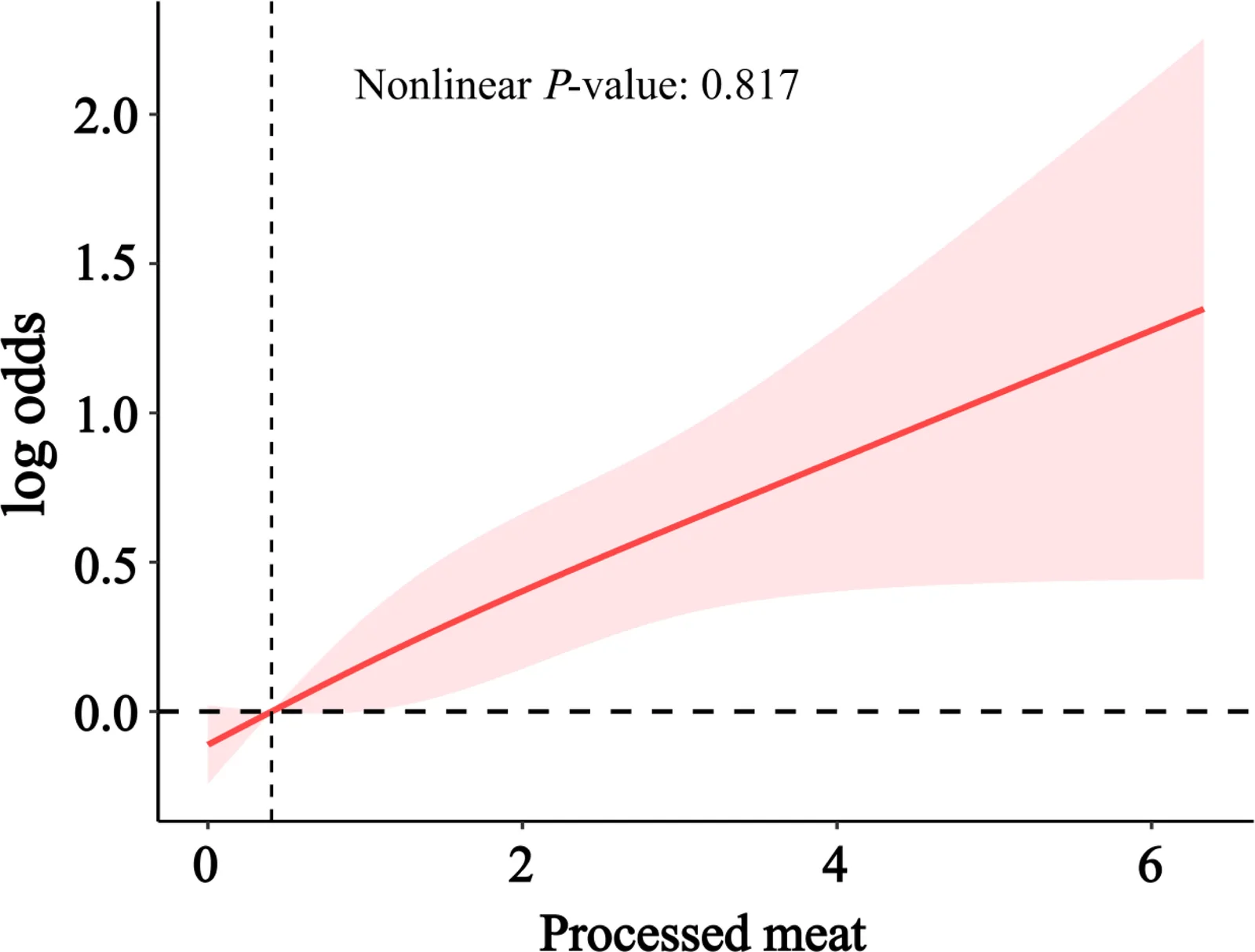
The fully adjusted relationship between continuous processed meat intake and metabolic dysfunction–associated steatotic liver disease using restricted cubic splines. The solid line represents the fitted nonlinear curve. The area adjacent to the solid line represents the 95% confidence interval.

To further characterize the association between continuous processed meat consumption and the odds of MASLD, a dose–response analysis using restricted cubic splines was performed (Figure 3). In the primary restricted spline model constructed with 3 knots, a positive linear dose–response relationship was observed after full adjustment for all potential confounders. Specifically, the odds of MASLD increased with higher daily processed meat intake, with no evidence of departure from linearity (*P* for nonlinearity = .817).

### Iso-serving Substitution Analysis

**Figure 4.**
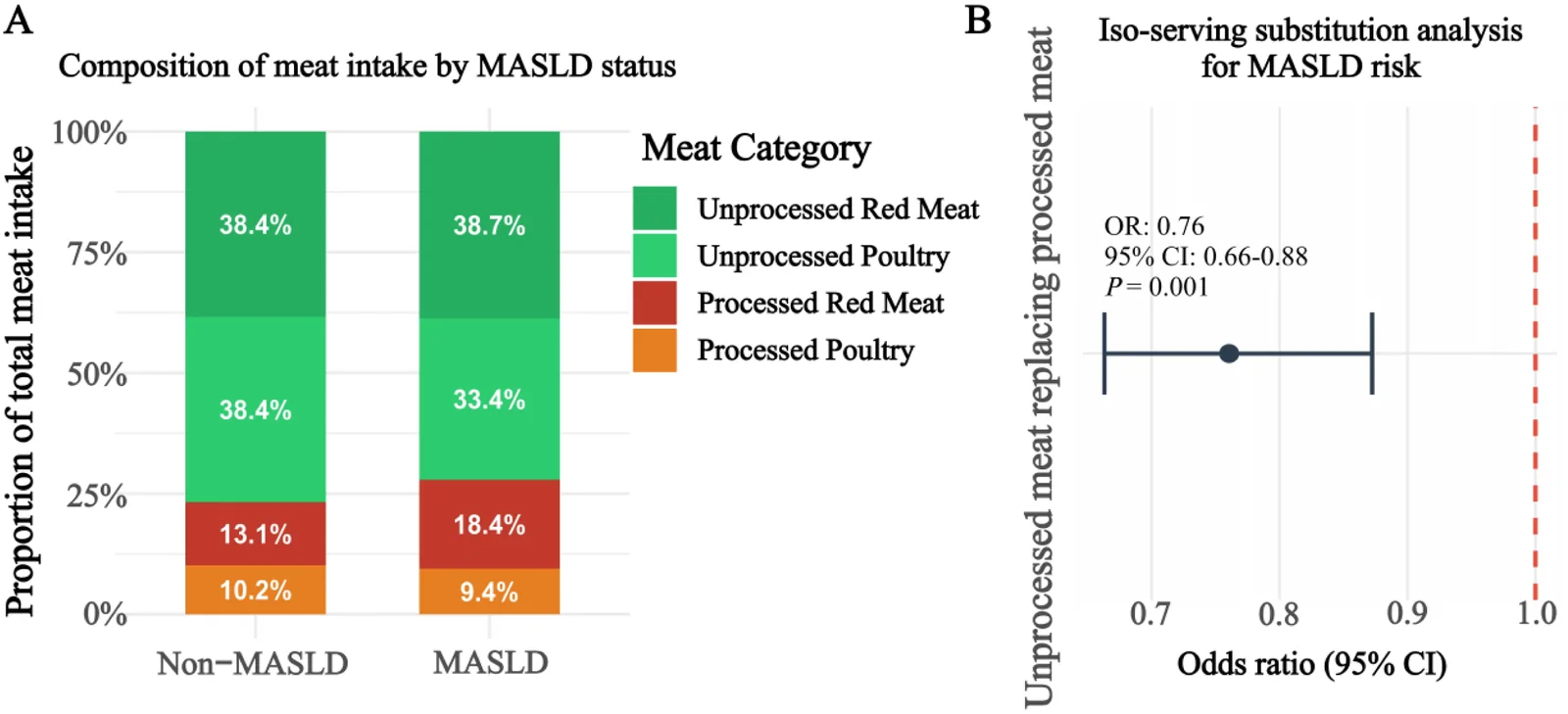
Composition of dietary meat intake and the theoretical iso-serving substitution effect on metabolic dysfunction–associated steatotic liver disease (MASLD) risk. (A) Weighted proportions of total meat intake contributed by unprocessed red meat, unprocessed poultry, processed red meat, and processed poultry, stratified by MASLD status. (B) Odds ratio (95% CI) for MASLD associated with a theoretical iso-serving substitution of 1 oz-eq/day of unprocessed meat for an equivalent amount of processed meat. The substitution model held total meat intake and total energy intake constant.

To visually contextualize these findings and explore potential dietary modification strategies, the proportional composition of meat intake stratified by MASLD status was first examined (Figure 4A). Descriptive analyses showed that processed meat (comprising both processed red meat and processed poultry) accounted for a higher proportion of total meat intake among individuals with MASLD compared with those without MASLD (27.8% vs. 23.3%).

Building on these dietary patterns, a theoretical iso-serving substitution analysis was conducted to model the potential implications of dietary substitution (Figure 4B). In the fully adjusted multivariable model, which held total meat intake and total daily energy intake constant, substituting 1 oz-eq/day of processed meat with an equivalent amount of unprocessed meat was significantly associated with 24% lower odds of MASLD (OR 0.76, 95% CI 0.66-0.88; *P* = .001). This finding suggests that substituting processed meat with unprocessed meat, while maintaining total meat intake, was associated with lower odds of MASLD.

## Discussion

The principal findings of this study demonstrate that higher consumption of total processed meat is independently and linearly associated with higher odds of MASLD, even after rigorous adjustment for a wide range of demographic, lifestyle, and metabolic confounders. Notably, this positive association was consistent across both disaggregated subtypes: processed red meat and processed poultry. In contrast, the total unprocessed meat intake, as well as its specific source subtypes (unprocessed red meat and unprocessed poultry), was not significantly associated with MASLD. Most importantly, theoretical iso-serving substitution analysis suggested that replacing processed meat with an equivalent amount of unprocessed meat was associated with lower odds of MASLD. Although the magnitude of the association between processed meat intake and MASLD was modest at the individual level, it may still be relevant at the population level, because both processed meat consumption and MASLD are common. At the same time, this effect size should be interpreted with caution and should not be considered comparable with that of major established metabolic risk factors. Together, these findings suggest that meat processing status, in addition to the total quantity consumed, may be an important dietary factor related to liver health.

Although some differences in the association between continuous processed meat intake and MASLD were observed across subgroups, including sex, all *P* for interaction values were >.05. Therefore, these findings should be interpreted with caution. In particular, the apparent sex-based difference may reflect underlying heterogeneity in hormonal status, body fat distribution, or metabolic responses, but it may also be attributable to limited statistical power or residual confounding. Further studies are needed to determine whether sex modifies this association.

The findings of the current study are consistent with and extend a growing body of epidemiological evidence suggesting that higher processed meat intake is adversely associated with fatty liver outcomes. The Mediterranean diet is currently recommended as a beneficial dietary approach for MASLD, and the current findings are compatible with this framework.^19^^,^^20^ In particular, the observed association with processed meat intake, together with the substitution analysis favoring unprocessed meat, is directionally consistent with dietary patterns characterized by lower processed meat, saturated fat, and cholesterol intake. In an Israeli population-based analysis with imaging-defined NAFLD, Zelber-Sagi and colleagues reported that higher consumption of red and processed meat was associated with NAFLD and insulin resistance, supporting the hypothesis that meat processing and related compounds may contribute to hepatic steatosis beyond total caloric intake.^21^ Similar patterns have been observed in ethnically diverse US populations. In the Multiethnic Cohort Study, higher intake of red and/or processed meat was associated with greater NAFLD risk, even after adjustment for major lifestyle and dietary factors.^22^ Importantly, most prior studies assessed “red and processed meat” jointly, relied on localized cohorts, or lacked adjustment for crucial lifestyle parameters. The current study extends this literature through several methodological strengths. First, the use of a nationally representative sample and adjustment for a comprehensive set of covariates—including total energy intake and physical activity—reduced the likelihood of residual confounding. Second, a standardized disaggregation algorithm was used to classify meat by both source and processing status. Crucially, after full multivariable adjustment, total unprocessed meat intake (combining fresh red meat and poultry) was not significantly associated with MASLD. Instead, the observed association was more evident for processed meats, with consistent findings for both processed red meat and processed poultry. This improved exposure specificity, coupled with the precision of CAP-defined MASLD, suggests that the artificial processing methods themselves, rather than the intrinsic nutritional profile or color of the meat, may be more relevant to the observed association.

Mechanistically, several components of processed meat may contribute to hepatic steatosis through converging metabolic pathways. Processed meats are typically high in sodium and contain preservatives such as nitrates and nitrites. Representative reference values for nitrate and nitrite and sodium content in commonly consumed processed meat products are available in published review articles.^23^^,^^24^ Experimental data suggest that nitrite-derived reactive nitrogen species can induce oxidative stress and mitochondrial dysfunction, thereby contributing to insulin resistance and hepatic lipid accumulation.^15^^,^^25^^-^^27^ High sodium intake has also been associated with systemic inflammation and metabolic dysregulation, potentially amplifying liver injury.^28^^,^^29^ In addition to oxidative stress and metabolic dysfunction, the gut microbiota may also contribute to the association between processed meat intake and MASLD. Meat consumption has been reported to influence gut microbial composition, and gut dysbiosis, together with disruption of the gut-liver axis, is increasingly recognized as an important mechanism in MASLD.^30^^,^^31^ Processed or preserved meat products are often rich in sodium, nitrites, and other additives, which may adversely affect the intestinal microbial environment and barrier function, thereby promoting hepatic inflammation and lipid accumulation. Experimental evidence also suggests that nitrite-containing processed meat may induce gut dysbiosis and broader metabolic disturbances.^32^ In addition, processed meats are a major source of heme iron.^33^ Excess heme iron can catalyze the formation of reactive oxygen species via Fenton chemistry, leading to lipid peroxidation and hepatocellular damage.^34^ Iron overload has been consistently associated with increased risk of steatosis and fibrosis progression in metabolic liver disease.^35^ Advanced glycation end products (AGEs), formed during high-temperature processing and curing, may further promote oxidative stress and inflammatory signaling through activation of the receptor for AGEs pathway.^36^ Saturated fatty acids, which are abundant in many processed meat products, have also been shown to induce endoplasmic reticulum stress and hepatic insulin resistance.^37^ Collectively, these mechanisms provide biologically plausible explanations for the observed associations in the study.

Beyond identifying risk associations, the iso-serving substitution analysis provides additional translational insight. In real-world settings, complete elimination of meat is often unrealistic and is associated with low long-term adherence. As shown in Figure 4, processed meat accounted for a greater proportion of total meat intake among participants with MASLD, and replacing processed meat with an equivalent amount of unprocessed meat was associated with lower odds of MASLD. These findings suggest that modifying the type of meat consumed, specifically trading processed meat with unprocessed alternatives, was associated with lower odds of MASLD without necessitating a reduction in total meat intake. Substitution modeling has emerged as an important methodological approach in nutritional epidemiology. Prior studies have demonstrated that replacing processed meat with fish or plant-based proteins was associated with a lower risk of type 2 diabetes and cardiovascular disease.^38,39^ By applying this framework to MASLD, the current study extends substitution analysis to liver outcomes and highlights its translational relevance. These findings may help inform dietary guidance emphasizing reduced processed meat intake as a feasible strategy to mitigate hepatometabolic risk.

The current study has several notable strengths. First, it used nationally representative NHANES data, enhancing generalizability to the US adult population. Second, MASLD was defined using CAP measured by ultrasound transient elastography, reducing outcome misclassification compared with surrogate indices. Third, the use of a standardized FPED-based algorithm enabled refined categorization of processed and unprocessed meat subtypes. The current study had several limitations. The cross-sectional design precludes causal inference and assessment of temporal relationships. Although the primary analyses were based on the average of 2 24-hour dietary recalls, dietary intake may not fully reflect long-term dietary patterns and remains susceptible to recall bias and underreporting. Although extensive covariates were adjusted for, residual confounding from unmeasured factors cannot be excluded. Because this study used US NHANES data, the findings may not be directly generalizable to non-Western populations or to regions with substantially different cultural dietary patterns, food processing practices, and background dietary structures. The supplementary analyses using CAP, LSM, and advanced fibrosis showed weaker or inconsistent patterns, suggesting that the observed association may be more closely related to steatosis-defined MASLD than to advanced disease severity. CAP-based MASLD primarily reflects hepatic steatosis and does not directly capture fibrosis stage or metabolic dysfunction-associated steatohepatitis (MASH) status.

In conclusion, findings from this nationally representative sample of US adults indicate that higher processed meat intake is independently associated with higher odds of MASLD. Furthermore, a theoretical substitution model suggested that replacing processed meat with an equivalent amount of unprocessed meat was associated with lower odds of MASLD. These findings may help inform future dietary recommendations aimed at reducing processed meat intake; however, confirmation in longitudinal and interventional studies is warranted.

## Supplementary Materials

Supplementary Material

## Figures and Tables

**Table 1.** Descriptive Characteristics of the Study Population Stratified by MASLD

Characteristic	Total N = 1828	Non-MASLD n = 1003	MASLD n = 825	*P*
Processed meat (lean oz-eq/day)	1.05 ± 0.06	0.94 ± 0.06	1.22 ± 0.10	**.032**
Processed red meat	0.64 ± 0.04	0.53 ± 0.05	0.81 ± 0.08	**.010**
Processed poultry	0.41 ± 0.03	0.41 ± 0.04	0.41 ± 0.04	.919
Unprocessed meat	3.12 ± 0.10	3.10 ± 0.15	3.16 ± 0.14	.758
Unprocessed red meat	1.61 ± 0.09	1.55 ± 0.10	1.70 ± 0.13	.343
Unprocessed poultry	1.51 ± 0.09	1.55 ± 0.12	1.47 ± 0.09	.553
Total energy intake (kcal/day)	2189 ± 42	2128 ± 58	2279 ± 46	**.043**
PA total MET (MET-min/week)	5723 ± 344	6279 ± 375	4902 ± 402	**.001**
TG (mmol/L)	1.52 ± 0.07	1.22 ± 0.05	1.97 ± 0.10	**<.001**
HDL (mmol/L)	1.40 ± 0.02	1.50 ± 0.02	1.24 ± 0.02	**<.001**
Age, n (%)				**<.001**
18-29	315 (20.89)	250 (82.34)	65 (17.66)	
30-44	444 (26.08)	288 (70.17)	156 (29.83)	
45-59	427 (25.25)	193 (48.44)	234 (51.56)	
≥60	642 (27.78)	272 (42.89)	370 (57.11)	
Sex, n (%)				**.003**
Female	894 (48.75)	529 (65.70)	365 (34.30)	
Male	934 (51.25)	474 (53.89)	460 (46.11)	
Race/ethnicity, n (%)				.106
Non‐Hispanic white	700 (66.98)	370 (60.66)	330 (39.34)	
Non‐Hispanic black	431 (10.27)	276 (65.92)	155 (34.08)	
Hispanic	331 (12.52)	160 (53.81)	171 (46.19)	
Other race	366 (10.23)	197 (53.89)	169 (46.11)	
Body mass index, n (%)				**<.001**
Underweight/normal	562 (31.89)	486 (92.32)	76 (7.68)	
Overweight	584 (30.69)	328 (61.40)	256 (38.60)	
Obese	682 (37.42)	189 (30.36)	493 (69.64)	
Marital status, n (%)				**<.001 **
Married/living with partner	1110 (63.43)	548 (53.02)	562 (46.98)	
Never married	357 (20.68)	267 (81.50)	90 (18.50)	
Widowed/divorced/separated	361 (15.89)	188 (57.66)	173 (42.34)	
Poverty income ratio, n (%)				.096
<1.3	441 (16.66)	266 (65.23)	175 (34.77)	
1.3-3.5	740 (34.57)	377 (54.86)	363 (45.14)	
>3.5	647 (48.77)	360 (61.13)	287 (38.87)	
Education levels, n (%)				0.656
Below high school	98 (2.14)	46 (52.83)	52 (47.17)	
High school	568 (31.63)	311 (58.69)	257 (41.31)	
Above high school	1162 (66.23)	646 (60.33)	516 (39.67)	
Alcohol consumption, n (%)				**<.001**
Never	206 (9.09)	99 (50.04)	107 (49.96)	
Former	391 (16.57)	150 (42.22)	241 (57.78)	
Mild	868 (50.22)	391 (47.75)	477 (52.25)	
Moderate	194 (12.72)	194 (100.00)	0 (0.00)	
Heavy	169 (11.40)	169 (100.00)	0 (0.00)	
Smoking status, n (%)				**.026**
Never	1123 (64.58)	610 (58.42)	513 (41.58)	
Former	438 (23.23)	198 (53.74)	240 (46.26)	
Now	267 (12.19)	195 (77.42)	72 (22.58)	
Hyperlipidemia, n (%)				**<.001**
No	613 (37.04)	458 (79.05)	155 (20.95)	
Yes	1215 (62.96)	545 (48.23)	670 (51.77)	
Hypertension, n (%)				**<.001**
No	1055 (66.05)	696 (69.85)	359 (30.15)	
Yes	773 (33.95)	307 (39.80)	466 (60.20)	
Diabetes mellitus, n (%)				**<.001**
No	1378 (81.24)	872 (67.34)	506 (32.66)	
Prediabetes	117 (6.42)	52 (37.45)	65 (62.55)	
DM	333 (12.34)	79 (20.51)	254 (79.49)	

Bold values in Table 1 indicate statistically significant results, defined as *P* < .05.

HDL, high-density lipoprotein; MASLD, metabolic dysfunction–associated steatotic liver disease; oz-eq/day, ounce-equivalents per day; PA total MET, total physical activity (MET-min/week); TG, triglycerides.

**Table 2.** Adjusted Association of Dietary Meat Intake with MASLD.

Exposure	Unadjusted Model	Adjust 1	Adjust 2
	Odds ratio (95% CI) Associated with MASLD; *P*		
Processed meat (continuous)	1.16 (1.03, 1.31); **.018**	1.22 (1.06, 1.40); **.012**	1.27 (1.10, 1.46); **.003**
Processed meat (tertiles)			
T1	1 (Ref)	1 (Ref)	1 (Ref)
T2	1.36 (0.90, 2.06); .128	1.54 (0.91, 2.63); .097	1.32 (0.64, 2.75); .425
T3	1.95 (1.26, 3.02); **.006**	2.30 (1.36, 3.88); **.006**	2.46 (1.48, 4.09); **.002**
Processed red meat (continuous)	1.32 (1.09, 1.61); **.009**	1.29 (1.05, 1.57); **.022**	1.23 (1.03, 1.48); **.028**
Processed red meat (tertiles)			
T1	1 (Ref)	1 (Ref)	1 (Ref)
T2	1.50 (1.00, 2.25); .052	1.59 (0.98, 2.58); 0.058	1.61 (0.88, 2.98); .116
T3	1.89 (1.32, 2.69); **.002**	2.02 (1.27, 3.22); **0.008**	1.53 (0.93, 2.50); .087
Processed poultry (continuous)	1.01 (0.89, 1.14); .919	1.15 (0.98, 1.35); 0.087	1.35 (1.10, 1.65); **.006**
Processed poultry (binary)			
0	1 (Ref)	1 (Ref)	1 (Ref)
>0	1.04 (0.78, 1.38); .780	1.28 (0.88, 1.85); .160	1.48 (0.94, 2.32); .082
Unprocessed meat (continuous)	1.01 (0.94, 1.08); .761	0.99 (0.93, 1.06); .734	0.95 (0.86, 1.05); .334
Unprocessed meat (tertiles)			
T1	1 (Ref)	1 (Ref)	1 (Ref)
T2	1.39 (0.92, 2.11); .110	1.33 (0.80, 2.21); .231	1.41 (0.77, 2.57); .248
T3	1.24 (0.81, 1.19); .291	1.10 (0.72, 1.67); .631	0.86 (0.49, 1.48); .557
Unprocessed red meat (continuous)	1.04 (0.96, 1.12); .344	1.00 (0.92, 1.09); .911	1.02 (0.92, 1.12); .747
Unprocessed red meat (tertiles)			
T1	1 (Ref)	1 (Ref)	1 (Ref)
T2	1.48 (0.95, 2.32); .081	1.44 (0.94, 2.19); .082	0.86 (0.48, 1.54); .592
T3	1.40 (0.98, 2.00); .065	1.22 (0.81, 1.83); .291	1.04 (0.62, 1.74); .886
Unprocessed poultry (continuous)	0.98 (0.92, 1.05); .510	0.98 (0.91, 1.05); .547	0.93 (0.81, 1.06); .262
Unprocessed poultry (tertiles)			
T1	1 (Ref)	1 (Ref)	1 (Ref)
T2	0.82 (0.62, 1.08); .144	0.83 (0.60, 1.16); .239	0.71 (0.42, 1.21); .194
T3	0.99 (0.76, 1.30); .959	1.03 (0.72, 1.47); .871	0.83 (0.49, 1.38); .445

Unadjusted model: nonadjusted model.

Adjust 1: Adjust for age, sex, and race.

Adjust 2: Adjust for age, sex, race, body mass index, poverty income ratio, education levels, marital status, PA total MET, total energy intake, smoking status, alcohol consumption, hyperlipidemia, hypertension, diabetes mellitus, triglycerides, and high-density lipoprotein.

Bold values in Table 2 indicate statistically significant results, defined as *P* < .05.

MASLD, metabolic dysfunction–associated steatotic liver disease; MET, total physical activity (MET-min/week); PA, physical activity.

**Table 3.** Sensitivity Analysis of the Association Between Dietary Meat Intake and MASLD Based on the First 24-Hour Dietary Recall

Exposure	Unadjusted Model	Adjust 1	Adjust 2
	Odds Ratio (95% CI) Associated with MASLD; *P*		
Processed meat (continuous)	1.08 (0.98, 1.19); .115	1.12 (0.99, 1.28);** .068**	1.12 (1.05, 1.37); **.011**
Processed meat (tertiles)			
T1	1 (Ref)	1 (Ref)	1 (Ref)
T2	1.20 (0.84, 1.73); .282	1.32 (0.84, 2.07); .195	1.27 (0.66, 2.44); .449
T3	1.65 (1.17, 2.33); **.007**	1.88 (1.22, 2.91); **.010**	2.18 (1.35, 3.51); **.003**
Processed red meat (continuous)	1.19 (1.05, 1.36); **.011**	1.18 (1.02, 1.37); **.034**	1.19 (1.01, 1.40); **.039**
Processed red meat (tertiles)			
T1	1 (Ref)	1 (Ref)	1 (Ref)
T2	1.35 (0.94, 1.94); .101	1.38 (0.89, 2.15); .133	1.51 (0.86, 2.64); .141
T3	1.62 (1.18, 2.24); **.006**	1.70 (1.15, 2.51); **.014**	1.46 (0.95, 2.24); .081
Processed poultry (continuous)	0.95 (0.85, 1.10); .564	1.08 (0.92, 1.27); .325	1.25 (1.00, 1.56); **.049**
Processed poultry (binary)			
0	1 (Ref)	1 (Ref)	1 (Ref)
>0	0.99 (0.72, 1.37); .968	1.20 (0.81, 1.77); .316	1.41 (0.84, 2.36); .179
Unprocessed meat (continuous)	1.01 (0.96, 1.08); .582	1.00 (0.94, 1.06); .919	0.95 (0.87, 1.04); .257
Unprocessed meat (tertiles)			
T1	1 (Ref)	1 (Ref)	1 (Ref)
T2	1.34 (0.90, 1.99); .137	1.24 (0.78, 1.96); .316	1.08 (0.64, 1.80); .765
T3	1.26 (0.88, 1.80); .188	1.10 (0.76, 1.60); .568	0.79 (0.48, 1.29); .322
Unprocessed red meat (continuous)	1.05 (0.98, 1.12); .165	1.02 (0.95, 1.10); .513	1.02 (0.92, 1.12); .739
Unprocessed red meat (tertiles)			
T1	1 (Ref)	1 (Ref)	1 (Ref)
T2	1.49 (0.96, 2.30); .072	1.46 (0.94, 2.28); .085	0.90 (0.47, 1.73); .745
T3	1.45 (1.01, 2.08); **.047**	1.27 (0.85, 1.89); .211	1.07 (0.62, 1.85); .805
Unprocessed poultry (continuous)	0.98 (0.93, 1.03); .333	0.97 (0.91, 1.04); .379	0.92 (0.82, 1.04); .185
Unprocessed poultry (tertiles)			
T1	1 (Ref)	1 (Ref)	1 (Ref)
T2	0.91 (0.65, 1.29); .590	0.93 (0.62, 1.38); .680	0.82 (0.44, 1.54); .518
T3	1.01 (0.77, 1.31); .951	1.03 (0.73, 1.44); .853	0.88 (0.52, 1.49); .613

Unadjusted model: nonadjusted model.

Adjust 1: Adjust for age, sex, and race.

Adjust 2: Adjust for age, sex, race, body mass index, poverty income ratio, education levels, marital status, PA total MET, total energy intake, smoking status, alcohol consumption, hyperlipidemia, hypertension, diabetes mellitus, triglycerides, and high-density lipoprotein.

MASLD, metabolic dysfunction–associated steatotic liver disease; MET, total physical activity (MET-min/week); PA, physical activity.

## Data Availability

The data that support the findings of this study are available on request from the corresponding author.
