# Supplementary material for: Processing Status, Rather Than Meat Source, Is Associated with Metabolic Dysfunction–Associated Steatotic Liver Disease Among US Adults
Source: Turk J Gastroenterol. 2026 Jul 9;37(9):972–84. doi: 10.5152/tjg.2026.26294 (PMC13554484; doi:10.5152/tjg.2026.26294)
Supplement: Supplementary file 1 [file supplementary_material.pdf]

Supplementary Table 1. Details of the categorization for dietary meat intake variables.

Variables	Division details	Corresponding range	Frequency	Percentage
Processed meat	T1	[0,0]	636	34.79%
	T2	[0, 1.015]	583	31.89%
	T3	[1.015,10.405]	609	33.32%
Processed red meat	T1	[0,0]	798	43.65%
	T2	[0,0.5]	423	23.14%
	T3	[0.5,8.885]	607	33.21%
Processed poultry	0	[0,0]	1354	74.07%
	>0	[0,7.07]	474	25.93%
Unprocessed meat	T1	[0,1.55]	611	33.42%
	T2	[1.55,3.6]	609	33.32%
	T3	[3.6,23.12]	608	33.26%
Unprocessed red meat	T1	[0,0]	615	33.64%
	T2	[0, 1.61]	604	33.04%
	T3	[1.61, 17.025]	609	33.32%
Unprocessed poultry	T1	[0, 0]	680	37.2%
	T2	[0, 1.83]	539	29.49%
	T3	[1.83, 16.405]	609	33.32%

Supplementary Table 2. The detailed overview of how we obtained information on hypertension, hyperlipidemia, and diabetes.

Hypertension	The inclusion of hypertension was determined by considering both the results of a questionnaire survey and three separate blood pressure measurements taken from the participants. The participants were asked the following questions: " {Have you/Has SP} ever been told by a doctor or other health professional that {you/s/he} had hypertension, also called high blood pressure? Because of {your/SP's} (high blood pressure/hypertension), {have you/has s/he} ever been told to . . . take prescribed medicine?" And Hypertension was also confirmed if the blood pressure measurements exceeded 140/90 mmHg.
Hyperlipidemia	The information regarding hyperlipidemia is primarily obtained based on the following criteria<br>1. High Triglycerides (TG): TG levels greater than or equal to 150 mg/dL.<br>2. High Total Cholesterol (TC): TC levels greater than or equal to 200 mg/dL [5.18 mmol/L].<br>3. Elevated Low-density lipoprotein (LDL)-Cholesterol: LDL cholesterol levels greater than or equal to 130 mg/dL [3.37 mmol/L].<br>4. Low high-density lipoprotein (HDL)-Cholesterol: In males, HDL cholesterol levels below 40 mg/dL [1.04 mmol/L]. In females, HDL cholesterol levels below 50 mg/dL [1.30 mmol/L].<br>5. The use of lipid-lowering medications.
Diabetes mellitus	The diagnostic criteria for diabetes are:<br>1. doctor told you have diabetes ({Other than during pregnancy, {have you/has SP}/ {Have you/Has SP}} ever been told by a doctor or health professional that {you have/{he/she/SP} has} diabetes or sugar diabetes?),<br>2. glycohemoglobin HbA1c (%) >= 6.5,<br>3. fasting glucose (mmol/l) >= 7.0,<br>4. random blood glucose (mmol/l) >= 11.1,<br>5. two-hour OGTT blood glucose (mmol/l) >= 11.1,<br>6. Use of diabetes medication or insulin,<br>Prediabetes: impaired fasting glycaemia and impaired glucose tolerance.

Supplementary Table 3. Sensitivity analysis of the association between dietary meat intake and MASLD after additional adjustment for Healthy Eating Index-2015 (HEI-2015).

	Unadjusted model	Model 1	Model 2 + HEI-2015
Exposure	Odds ratio (95% confidence interval) associated with MASLD; *P*-value		
Processed meat (continuous)	1.16 (1.03, 1.31); **0.018**	1.22 (1.06, 1.40); **0.012**	1.25 (1.09, 1.44); **0.004**
Processed meat (tertiles)			
T1	1 (Ref)	1 (Ref)	1 (Ref)
T2	1.36 (0.90, 2.06); 0.128	1.54 (0.91, 2.63); 0.097	1.29 (0.64, 2.63); 0.451
T3	1.95 (1.26, 3.02); **0.006**	2.30 (1.36, 3.88); **0.006**	2.37 (1.47, 3.85); **0.002**
Processed red meat (continuous)	1.32 (1.09, 1.61); **0.009**	1.29 (1.05, 1.57); **0.022**	1.21 (1.01, 1.45); **0.043**
Processed red meat (tertiles)			
T1	1 (Ref)	1 (Ref)	1 (Ref)
T2	1.50 (1.00, 2.25); 0.052	1.59 (0.98, 2.58); 0.058	1.57 (0.88, 2.83); 0.119
T3	1.89 (1.32, 2.69); **0.002**	2.02 (1.27, 3.22); **0.008**	1.45 (0.90, 2.34); 0.114
Processed poultry (continuous)	1.01 (0.89, 1.14); 0.919	1.15 (0.98, 1.35); 0.087	1.34 (1.11, 1.62); **0.005**
Processed poultry (binary)			
0	1 (Ref)	1 (Ref)	1 (Ref)
>0	1.04 (0.78, 1.38); 0.780	1.28 (0.88, 1.85); 0.160	1.46 (0.93, 2.28); 0.091
Unprocessed meat (continuous)	1.01 (0.94, 1.08); 0.761	0.99 (0.93, 1.06); 0.734	0.96 (0.86, 1.06); 0.349
Unprocessed meat (tertiles)			
T1	1 (Ref)	1 (Ref)	1 (Ref)
T2	1.39 (0.92, 2.11); 0.110	1.33 (0.80, 2.21); 0.231	1.40 (0.77, 2.54); 0.248
T3	1.24 (0.81, 1.19); 0.291	1.10 (0.72, 1.67); 0.631	0.86 (0.50, 1.49); 0.574
Unprocessed red meat (continuous)	1.04 (0.96, 1.12); 0.344	1.00 (0.92, 1.09); 0.911	1.01 (0.92, 1.11); 0.837
Unprocessed red meat (tertiles)			
T1	1 (Ref)	1 (Ref)	1 (Ref)
T2	1.48 (0.95, 2.32); 0.081	1.44 (0.94, 2.19); 0.082	0.84 (0.47, 1.50); 0.531
T3	1.40 (0.98, 2.00); 0.065	1.22 (0.81, 1.83); 0.291	1.01 (0.61, 1.67); 0.967
Unprocessed poultry (continuous)	0.98 (0.92, 1.05); 0.510	0.98 (0.91, 1.05); 0.547	0.93 (0.81, 1.07); 0.309
Unprocessed poultry (tertiles)			
T1	1 (Ref)	1 (Ref)	1 (Ref)
T2	0.82 (0.62, 1.08); 0.144	0.83 (0.60, 1.16); 0.239	0.69 (0.41, 1.17); 0.157
T3	0.99 (0.76, 1.30); 0.959	1.03 (0.72, 1.47); 0.871	0.84 (0.50, 1.42); 0.449

Unadjusted model: non-adjusted model.
Model 1: Adjust for age, sex, race.
Model 2 + HEI-2015: Adjust for age, sex, race, body mass index, poverty income ratio, education levels, marital status, smoking status, alcohol consumption, PA total MET, hyperlipidemia, hypertension, diabetes mellitus, total energy intake, HEI-2015, triglycerides and high-density lipoprotein.
HEI-2015, Healthy Eating Index-2015; MASLD, Metabolic dysfunction-associated steatotic liver disease.

Supplementary Table 4. Supplementary analysis of the association between dietary meat intake and controlled attenuation parameter (CAP) after additional adjustment for Healthy Eating Index-2015 (HEI-2015).

	Unadjusted model	Model 1	Model 2 + HEI-2015
Exposure	β (95% CI) associated; *P*-value		
Processed meat (continuous)	2.07 (-0.86, 5.01); 0.151	2.17 (-1.04, 5.39); 0.161	-0.54 (-2.57, 1.49); 0.580
Processed meat (tertiles)			
T1	1 (Ref)	1 (Ref)	1 (Ref)
T2	8.03 (-0.84, 16.89); 0.072	9.56 (-1.04, 20.16); 0.071	1.45 (-4.64, 7.54); 0.619
T3	12.95 (2.81, 23.09); **0.016**	13.21 (3.45, 22.97); **0.014**	2.05 (-4.90, 9.00); 0.540
Processed red meat (continuous)	5.87 (1.61, 10.12); **0.010**	3.76 (-0.15, 7.68); 0.058	-0.44 (-3.26, 2.39); 0.747
Processed red meat (tertiles)			
T1	1 (Ref)	1 (Ref)	1 (Ref)
T2	10.87 (-1.73, 23.47); 0.085	10.75 (-3.45, 24.96); 0.119	2.32 (-5.72, 10.36); 0.547
T3	14.12 (4.45, 23.80); **0.008**	12.17 (2.85, 21.48); **0.017**	-0.57 (-6.43, 5.28); 0.837
Processed poultry (continuous)	-2.46 (-6.17, 1.25); 0.177	0.38 (-4.18, 4.94); 0.854	-0.68 (-3.34, 1.97); 0.591
Processed poultry (binary)			
0	1 (Ref)	1 (Ref)	1 (Ref)
>0	-0.88 (-10.40, 8.65); 0.846	3.95 (-6.31, 14.21); 0.407	1.02 (-6.05, 8.09); 0.762
Unprocessed meat (continuous)	0.85 (-1.35, 3.04); 0.423	0.20 (-1.73, 2.12); 0.823	-0.24 (-1.36, 0.87); 0.647
Unprocessed meat (tertiles)			
T1	1 (Ref)	1 (Ref)	1 (Ref)
T2	13.7 (2.23, 25.17); **0.023**	10.99 (-1.39, 23.36); 0.075	7.23 (-2.15, 16.60); 0.121
T3	12.43 (-1.96, 26.81); 0.085	7.56 (-5.23, 20.36); 0.210	1.91 (-4.99, 8.82); 0.564
Unprocessed red meat (continuous)	2.46 (0.15, 4.77); **0.039**	1.30 (-0.60, 3.19); 0.156	0.98 (-0.03, 1.99); 0.056
Unprocessed red meat (tertiles)			
T1	1 (Ref)	1 (Ref)	1 (Ref)
T2	15.46 (1.56, 29.35); **0.032**	12.88 (1.23, 24.53); **0.034**	0.87 (-8.23, 9.98); 0.841
T3	18.2 (6.73, 29.68); **0.005**	12.62 (2.89, 22.35); **0.017**	5.54 (-1.19, 12.27); 0.100
Unprocessed poultry (continuous)	-0.83 (-2.69, 1.04); 0.357	-0.84 (-2.82, 1.13); 0.358	-1.22 (-2.63, 0.19); 0.085
Unprocessed poultry (tertiles)			
T1	1 (Ref)	1 (Ref)	1 (Ref)
T2	-8.16 (-16.19, -0.12); **0.047**	-6.57 (-15.33, 2.18); 0.122	-10.7 (-16.69, 4.71); 0.302
T3	-2.73 (-9.96, 4.51); 0.430	-1.79 (-10.99, 7.42); 0.667	-4.83 (-10.48, 0.81); 0.088

CAP was analyzed as a continuous outcome (dB/m).
Unadjusted model: non-adjusted model.
Model 1: Adjust for age, sex, race.
Model 2 + HEI-2015: Adjust for age, sex, race, body mass index, poverty income ratio, education levels, marital status, smoking status, alcohol consumption, PA total MET, hyperlipidemia, hypertension, diabetes mellitus, total energy intake, HEI-2015, triglycerides and high-density lipoprotein.
CAP, Controlled attenuation parameter; HEI-2015, Healthy Eating Index-2015; MASLD, Metabolic dysfunction–associated steatotic liver disease.

Supplementary Table 5. Supplementary analysis of the association between dietary meat intake and liver stiffness measurement after additional adjustment for Healthy Eating Index-2015 (HEI-2015).

	Unadjusted model	Model 1	Model 2 + HEI-2015
Exposure	β (95% CI) associated; *P*-value		
Processed meat (continuous)	-0.1 (-0.30, 0.10); 0.310	-0.11 (-0.30, 0.09); 0.242	-0.23 (-0.47, 0.00); 0.053
Processed meat (tertiles)			
T1	1 (Ref)	1 (Ref)	1 (Ref)
T2	-0.64 (-1.37, 0.08); 0.076	-0.62 (-1.40, 0.16); 0.106	-0.67 (-1.33, 0.02); 0.056
T3	-0.54 (-1.45, 0.38); 0.229	-0.57 (-1.55, 0.41); 0.218	-0.94 (-1.97, 0.10); 0.073
Processed red meat (continuous)	0 (-0.29, 0.28); 0.972	-0.06 (-0.34, 0.21); 0.613	-0.26 (-0.64, 0.12); 0.167
Processed red meat (tertiles)			
T1	1 (Ref)	1 (Ref)	1 (Ref)
T2	-0.78 (-1.48, -0.07); **0.033**	-0.79 (-1.57, -0.01); **0.047**	-0.86 (-1.51, 0.03); 0.056
T3	-0.41 (-1.28, 0.47); 0.334	-0.47 (-1.38, 0.44); 0.264	-0.82 (-1.79, 0.15); 0.092
Processed poultry (continuous)	-0.23 (-0.43, -0.03); **0.028**	-0.17 (-0.37, 0.04); 0.097	-0.22 (-0.43, 0.09); 0.103
Processed poultry (binary)			
0	1 (Ref)	1 (Ref)	1 (Ref)
>0	-0.55 (-1.01, -0.10); **0.021**	-0.45 (-0.95, 0.04); 0.067	-0.68 (-1.21, 0.11); 0.117
Unprocessed meat (continuous)	0.14 (0.00, 0.28); 0.057	0.13 (-0.02, 0.27); 0.080	0.09 (-0.03, 0.21); 0.134
Unprocessed meat (tertiles)			
T1	1 (Ref)	1 (Ref)	1 (Ref)
T2	-0.28 (-0.86, 0.30); 0.315	-0.34 (-0.97, 0.29); 0.246	-0.48 (-0.96, 0.00); 0.050
T3	0.55 (-0.16, 1.25); 0.118	0.45 (-0.27, 1.17); 0.191	0.19 (-0.39, 0.77); 0.493
Unprocessed red meat (continuous)	0.22 (-0.02, 0.46); 0.073	0.20 (-0.05, 0.46); 0.104	0.15 (-0.06, 0.35); 0.151
Unprocessed red meat (tertiles)			
T1	1 (Ref)	1 (Ref)	1 (Ref)
T2	0.43 (-0.16, 1.01); 0.140	0.38 (-0.19, 0.96); 0.163	0.04 (-0.43, 0.51); 0.860
T3	0.86 (0.16, 1.57); **0.020**	0.77 (-0.02, 1.55); 0.054	0.40 (-0.23, 1.04); 0.197
Unprocessed poultry (continuous)	0.03 (-0.13, 0.19); 0.719	0.02 (-0.14, 0.18); 0.808	0.01 (-0.15, 0.16); 0.933
Unprocessed poultry (tertiles)			
T1	1 (Ref)	1 (Ref)	1 (Ref)
T2	-0.29 (-1.15, 0.56); 0.474	-0.26 (-1.13, 0.62); 0.516	-0.35 (-1.10, 0.40); 0.338
T3	-0.20 (-0.94, 0.54); 0.564	-0.23 (-0.97, 0.51); 0.494	-0.20 (-0.85, 0.45); 0.521

Liver stiffness measurement was analyzed as a continuous outcome (kPa).
Unadjusted model: non-adjusted model.
Model 1: Adjust for age, sex, race.
Model 2 + HEI-2015: Adjust for age, sex, race, body mass index, poverty income ratio, education levels, marital status, smoking status, alcohol consumption, PA total MET, hyperlipidemia, hypertension, diabetes mellitus, total energy intake, HEI-2015, triglycerides and high-density lipoprotein.
HEI-2015, Healthy Eating Index-2015; MASLD, Metabolic dysfunction–associated steatotic liver disease.

Supplementary Table 6. Supplementary analysis of the association between dietary meat intake and advanced fibrosis after additional adjustment for Healthy Eating Index-2015 (HEI-2015).

	Unadjusted model	Model 1	Model 2 + HEI-2015
Exposure	Odds ratio (95% confidence interval) associated with advanced fibrosis; *P*-value		
Processed meat (continuous)	0.82 (0.60, 1.12); 0.190	0.80 (0.55, 1.15); 0.194	0.65 (0.45, 0.93); **0.022**
Processed meat (tertiles)			
T1	1 (Ref)	1 (Ref)	1 (Ref)
T2	0.52 (0.19, 1.43); 0.187	0.52 (0.17, 1.59); 0.215	0.50 (0.21, 1.19); 0.108
T3	0.53 (0.18, 1.53); 0.215	0.48 (0.14, 1.68); 0.215	0.30 (0.10, 0.94); **0.041**
Processed red meat (continuous)	0.86 (0.61, 1.22); 0.374	0.81 (0.53, 1.23); 0.274	0.66 (0.43, 1.02); 0.060
Processed red meat (tertiles)			
T1	1 (Ref)	1 (Ref)	1 (Ref)
T2	0.46 (0.17, 1.19); 0.101	0.44 (0.15, 1.27); 0.111	0.36 (0.13, 0.96); **0.042**
T3	0.56 (0.23, 1.34); 0.173	0.49 (0.18, 1.39); 0.154	0.35 (0.12, 1.01); 0.052
Processed poultry (continuous)	0.72 (0.42, 1.24); 0.218	0.76 (0.44, 1.31); 0.283	0.57 (0.29, 1.12); 0.098
Processed poultry (binary)			
0	1 (Ref)	1 (Ref)	1 (Ref)
>0	0.54 (0.20, 1.41); 0.188	0.57 (0.20, 1.60); 0.251	0.32 (0.12, 0.88); **0.030**
Unprocessed meat (continuous)	1.16 (1.05, 1.28); **0.006**	1.18 (1.05, 1.33); **0.011**	1.21 (1.11, 1.31); **< 0.001**
Unprocessed meat (tertiles)			
T1	1 (Ref)	1 (Ref)	1 (Ref)
T2	0.66 (0.36, 1.22); 0.165	0.64 (0.32, 1.26); 0.167	0.56 (0.33, 0.98); **0.043**
T3	2.72 (1.26, 5.88); **0.015**	2.85 (1.21, 6.69); **0.022**	2.92 (1.37, 6.19); **0.008**
Unprocessed red meat (continuous)	1.23 (1.02, 1.50); **0.035**	1.24 (1.01, 1.53); **0.046**	1.23 (1.01, 1.50); **0.042**
Unprocessed red meat (tertiles)			
T1	1 (Ref)	1 (Ref)	1 (Ref)
T2	1.42 (0.67, 3.01); 0.327	1.36 (0.63, 2.95); 0.388	1.02 (0.53, 1.98); 0.944
T3	2.53 (0.93, 6.85); 0.065	2.41 (0.82, 7.04); 0.096	2.00 (0.66, 6.10); 0.203
Unprocessed poultry (continuous)	1.07 (0.91, 1.25); 0.396	1.07 (0.90, 1.27); 0.400	1.10 (0.96, 1.25); 0.156
Unprocessed poultry (tertiles)			
T1	1 (Ref)	1 (Ref)	1 (Ref)
T2	0.68 (0.26, 1.78); 0.398	0.69 (0.26, 1.81); 0.403	0.67 (0.27, 1.65); 0.359
T3	1.04 (0.36, 2.99); 0.943	1.05 (0.35, 3.14); 0.926	1.44 (0.58, 3.57); 0.409

Advanced fibrosis was defined as liver stiffness measurement ≥ 9.7 kPa.
Unadjusted model: non-adjusted model.
Model 1: Adjust for age, sex, race.
Model 2 + HEI-2015: Adjust for age, sex, race, body mass index, poverty income ratio, education levels, marital status, smoking status, alcohol consumption, PA total MET, hyperlipidemia, hypertension, diabetes mellitus, total energy intake, HEI-2015, triglycerides and high density lipoprotein.
HEI-2015, Healthy Eating Index-2015; MASLD, Metabolic dysfunction–associated steatotic liver disease.
